# Effect of Cultivar Resistance and Soil Management on Spatial–Temporal Development of *Verticillium* Wilt of Olive: A Long-Term Study

**DOI:** 10.3389/fpls.2020.584496

**Published:** 2020-10-27

**Authors:** Eduardo Ostos, María Teresa Garcia-Lopez, Rafael Porras, Francisco J. Lopez-Escudero, Antonio Trapero-Casas, Themis J. Michailides, Juan Moral

**Affiliations:** ^1^Department of Agronomy, University of Córdoba, Córdoba, Spain; ^2^Department of Plant Pathology, University of California, Davis, Davis, CA, United States; ^3^Biogeos, Estudios Ambientales, Centauro, Spain

**Keywords:** *Verticillium* wilt, Integrated Disease Management, olive, *Verticillium*, plant pathogen control

## Abstract

*Verticillium* wilt, caused by *Verticillium dahliae*, challenges olive cultivation and an Integrated Disease Management (IDM) approach is the best-suited tool to combat it. Since 1998, an IDM strategy in an orchard (called Granon, Spain) of the susceptible cv. Picual was conducted by increasing planting density with moderately resistant cv. Frantoio, chemical weed control, and replanting of dead olives with cv. Frantoio following soil solarization. The *Verticillium* wilt epidemic in Granon orchard was compared to the epidemic in a non-IDM orchard (called Ancla, Spain) with plowed soil and dead Picual olives replanted with the same cultivar. Field evaluations (2012–2013) showed an incidence and severity of the disease as Picual–Ancla > Picual–Granon > Frantoio–Granon. The spatiotemporal dynamics of the *Verticillium* epidemics from 1998 to 2010 were monitored with digital images using SIG. The annual tree mortalities were 5.6% for Picual olives in Ancla orchard, and 3.1 and 0.7% for Picual and Frantoio olives in Granon orchard, respectively. There was a negative relationship between the mortality of olive trees (%) by the pathogen and the height (m) above sea level. The annual mortality of cv. Picual olives was positively correlated with spring rainfalls. The Index of Dispersion and beta-binomial distribution showed aggregation of *Verticillium*-dead olives. In conclusion, this IDM strategy considerably reduced the disease in comparison with traditional agronomic practices.

## Introduction

The olive tree (*Olea europaea* L. subsp. *europaea*) is one of the most important woody crops around the world. Spain accounts for the largest production of both olive oil and table fruit. In this country, the major area of olive groves covers around 1.65 million hectares and is located in Andalusia, Southern region, being one-third concentrated in the province of Jaen (ESYRCE, 2019). *Verticillium* wilt of olive, caused by the soil-borne pathogen *Verticillium dahliae* Kleb., is a major concern for the producers of this crop worldwide (Lopez-Escudero and Mercado-Blanco, 2011; Tsror, 2011; Keykhasaber et al., 2017). In Spain, the increase and spread of *Verticillium* wilt of olive have been mainly driven by (i) the olive colonization of soils historically cropped with cotton, which is a major host of *V. dahliae*; and (ii) the spread of the defoliating (D) pathotype of the pathogen, which is more virulent than the previously dominant non-defoliating pathotype (ND) (Navas-Cortes et al., 2008; Lopez-Escudero et al., 2010; Milgroom et al., 2016). The D isolates of *V. dahliae* cause defoliation in olive and cotton, whereas ND isolates cause wilting but no defoliation (Lopez-Escudero et al., 2004; Jimenez-Diaz et al., 2017).

In the disease cycle of *Verticillium* wilt of olive, the pathogen has a parasitic phase in the host and a non-parasitic phase as microsclerotia (Keykhasaber et al., 2017), which can survive in the field soils for a prolonged period (up to 15 years) (Wilhelm, 1955). Microsclerotia germinate under favorable microbiological and environmental conditions and in the presence of root exudates of olive infect the roots (Prieto et al., 2009; Jimenez-Diaz et al., 2012). Subsequently, the pathogen systemically colonizes the olive tree with hyphae and conidia before symptoms develop (Jimenez-Diaz et al., 2012). Colonized trees show characteristic canopy wilting and ultimately die (Navas-Cortes et al., 2008). However, some infected olive trees can overcome the disease (Levin et al., 2003; Lopez-Escudero and Blanco-Lopez, 2005; Bubici and Cirulli, 2014).

Because no control measures applied singly are completely effective in controlling *Verticillium* wilt of olive, the disease should be managed according to an Integrated Disease Management (IDM) strategy (Tsror, 2011; Jimenez-Diaz et al., 2012). An IDM strategy should be based on the application of both preplanting and postplanting control measures (Lopez-Escudero and Mercado-Blanco, 2011). Preplanting control measures include (i) the quantification of the pathogen in soil while avoiding planting in infested soils (Perez-Artes et al., 2005); (ii) the use of pathogen-free planting material (Morello et al., 2016); and (iii) the selection of cultivars with elevated resistance to the *V. dahliae*, because there are no immune ones (Lopez-Escudero et al., 2004; Trapero et al., 2013; Leyva-Perez et al., 2017). After planting, control measures may include (i) the elimination of alternative weed hosts to avoid the increase in pathogen inoculum (Ligoxigakis et al., 2002); (ii) the removal of pruning remains by burning (Morello et al., 2016); (iii) the application of disinfectants to reduce the viability of the pathogen in the irrigation water (Gomez-Galvez et al., 2018; Gomez-Galvez and Rodriguez-Jurado, 2018); or (iv) biofumigation by sowing Brassicaceae species and/or solarization because both strategies reduce pathogen inoculum density in the soil (Tjamos, 1991; Lopez-Escudero and Blanco-Lopez, 2001; Bejarano-Alcazar, 2008). The use of an IDM strategy to control *Verticillium* wilt of olive has been highly recommended for many authors (Lopez-Escudero and Mercado-Blanco, 2011; Jimenez-Diaz et al., 2012; Keykhasaber et al., 2017; Montes-Osuna and Mercado-Blanco, 2020); however, the examination of an IDM strategy controlling the disease in a commercial olive orchard and the long-term effect in field conditions is needed. While spatiotemporal studies have highlighted their potential in understanding epidemics of soil-borne pathogens (Madden et al., 2007), these remain barely used in *Verticillium* wilt of olive and limited to the moderately susceptible cultivar Arbequina in experimental plots (Navas-Cortes et al., 2008). Generally, olive trees showing *Verticillium* wilt symptoms occur in clusters in the field where the disease is driven by the natural inoculum, i.e., the soil microsclerotia (Navas-Cortes et al., 2008), although the use of infected planting stocks could also contribute to increase the heterogeneity of affected olives in the field (Thanassoulopoulos, 1992). The effect of *Verticillium* wilt management practices on the spatial patterns of dead olives by the pathogen will aid in the understanding of the impact of these control methods on the diseases and how we can improve them.

Thereunder, we conducted an epidemiological study in two commercial olive orchards intending to characterize the impact of a specific IDM strategy on spatiotemporal of *V. dahliae–*dead olives for 12 years. The IDM described in the present study has contributed to keeping with relatively low levels of *Verticillium* wilt in a commercial olive orchard.

## Materials and Methods

### Orchards and Background

The present study was conducted in two adjacent (distance between them 100 m) commercial olive groves (4159393.6N, 457616.1W), called Ancla and Granon, counting 21.5 and 16.2 ha, respectively. Both Ancla and Granon orchards stand 329 m (from 264 to 384 m) and 286 m (from 260 to 312 m) above sea level. The orchards were separated by an 18-m-wide road and located in the province of Jaen, in Andalusia (South of Spain), considered to be the leading olive-growing region in the world, having more than 592,000 ha of this crop (ESYRCE, 2019). This location held an average rainfall of 524 mm and average temperature of 17°C, and a xerofluvent soil, according to Soil Survey Staff (2015). Both orchards originally belonged to the same owner, and they were planted with *V. dahliae–*susceptible herbaceous crops (alfalfa, cotton, and tomato) for 25 years. Then, the ownership of the orchards was transferred to two olive farmers, just before the starting of the study.

For planting, 1-year-old self-rotten olives cv. Picual were used, which are highly susceptible to both non-defoliating and defoliating pathotypes of the pathogen (Lopez-Escudero et al., 2004; Roca et al., 2016). The olives were established during the fall–winter of 1995–1996 (Ancla orchard) and the fall–winter of 1996–1997 (Granon orchard). In Granon orchard, the spacing was 10 × 10 m^2^ (100 trees per hectare). In Ancla orchard, the tree spacing was the same (10 × 10 m^2^), although 11 rows of olive trees were planted at 6 × 3 m^2^ on the west side of the orchard.

We observed the first *Verticillium* wilt symptoms in spring 1998, 2 years after plantation, in Ancla orchard. In 2000, the pathogen was isolated on potato dextrose agar (PDA) of 10 symptomatic trees of each orchard as described below. Also, inoculum densities of *V. dahliae* microsclerotia on the soil of both orchards were estimated by subsampling using the wet sieving technique as described below (*Soil Isolation*). At this point, the inoculum density in both orchards was similar and ranged from 0.4 to 0.6 microsclerotia per gram of soil. Also, in 2010, an evaluation of *Phytophthora* species was performed, but with no detection of the pathogen (Moral, unpublished data).

### Agronomic Practices

#### Granon Orchard

From 1998, the agronomic practices applied to the Granon orchard aimed to decrease the *Verticillium* impact. At that time, we started an IDM strategy based on the combination of diverse control methods that had individually shown their efficacy controlling the pathogen. This IDM strategy has been systematically applied since 1998 and is based on (i) weed control by herbicides (mainly glyphosate) to avoid inoculum production of the pathogen on alternative hosts (Thanassoulopoulos et al., 1981); (ii) burning of the pruning debris to prevent the spread of *V. dahliae* inoculum (Morello et al., 2016); (iii) annual harvesting of the fallen leaves of the symptomatic olive trees using leaf blowers and eliminating through burning; (iv) uprooting, burning, and solarizing the soil by using a polyethylene sheet (4 × 4 m^2^) for 50–60 days during the summer (Lopez-Escudero and Blanco-Lopez, 2001); and finally (v) during the fall, the moderately resistant cv. Frantoio was used to replant the solarized sites to replace the dead tree (Lopez-Escudero et al., 2004; Leyva-Perez et al., 2017). In 2000, an olive tree of the moderately resistant cv. Frantoio was planted in the middle of every four olive trees of the cv. Picual increasing the tree density from 100 to 204 olive trees ha^–1^. Also, a drip irrigation system was installed, consisting of two drippers (8 L h^–1^) per tree and 1,500 m^3^ ha^–1^ yr^–1^. With variations from one season to another, irrigation was mainly applied during spring and autumn (approximately 80% of the total applied water), whereas more severe water restrictions were applied during the summer. Furthermore, despite that infective propagules of *V. dahliae* are common in irrigation waters in Andalusia (Garcia-Cabello et al., 2012), no water surveys were conducted to test the presence of the pathogen in the water, and subsequently, no specific management was conducted in this sense.

#### Ancla Orchard

In this orchard, the management followed the traditional agronomic practices performed by olive growers in Jaen province. The Ancla orchard was rainfed, and the dead olives of cv. Picual were replaced with trees belonging to the same susceptible Picual cultivar. The topsoil layer was mechanically tilled for weed control, and pruning debris were burned.

In both Granon and Ancla orchards, different copper-based fungicides were used within a normal annual treatment schedule (two to three treatments per year) during spring and fall–winter to control different aerial diseases such as *Venturia oleaginea* and *Pseudocercospora cladosporioides* (Moral et al., 2018). These compounds have no activity against vascular diseases such as *Verticillium* wilt (Fodale and Mule, 1999).

### Weather Data

Weather data were obtained from the nearby (∼10 km) Linares weather station (443,002.0 N, 4,212,540.0 W, altitude 432 m). Weather data collection included daily measurements of temperature and precipitation. Therefore, we calculated the monthly data for the following parameters: minimum, maximum, and average temperatures and total rainfall. Similarly, we calculated the same parameters for each season, i.e., fall (September–November), winter (December–February), spring (March–May), and summer (June–August).

### Field Evaluations

Field evaluations were conducted in 2012 and 2013. For that, we chose three experimental plots in Granon orchard with around 200 olive trees per plot (100 of each, Frantoio and Picual cultivars). Because of the very high disease intensity in the Ancla orchard, we only defined an experimental plot with 200 olive trees cv. Picual in the center of the orchard. All the olive tree positions were marked according to the row number and within the row tree number.

#### Disease Parameters

The symptoms, in terms of incidence and disease severity, were evaluated in June 2012, October 2012, April 2013, June 2013, and October 2013. The disease severity was assessed with a 0–6-point rating scale where 0 = no symptoms, 1% < 5%, 2 = 5–10%, 3 = 11–20%, 4 = 21–40%, 5 = 41–80% of wilted canopy, 6 = dead tree. We used this rating scale since affected olive trees usually showed a low percentage of wilted canopy, while those showing more than 80% of their canopy wilted were dead. Each tree was gauged by two evaluators, and the mean value of the evaluations was used for the data analysis. The rating scale values helped to calculate (i) the Disease Intensity Index (DII), as the average severity of all the trees (including the no affected ones); ii) the disease severity, as the average severity of all the affected trees; and (iii) dead trees percentage or mortality.

#### Pathogen Isolation and Identification

During every field evaluation, wilted shoot samples were taken from 15 (Granon orchard, *n* = 75) and 10 (Ancla orchard, *n* = 50) symptomatic olive trees cv. Picual to conduct the isolation of the pathogen on acidified PDA culture media (Difco Laboratories, Detroit, MI, United States) (Lopez-Escudero et al., 2004). Similarly, isolations from samples of 10 asymptomatic and two symptomatic (*n* = 12) trees of the olive cv. Frantoio were conducted.

#### Pathogen Detection by Quantitative Polymerase Chain Reaction

A previously validated real-time polymerase chain reaction (PCR) protocol with the specific primers VertBt-F and VertBt-R (Atallah et al., 2007) was used (Morello et al., 2016). DNA was isolated using the DNeasy Plant Mini kit (QIAGEN). The quantitative PCR (qPCR) was conducted using SYBR Green PCR Master Mix (Applied Biosystems) using an iCycler (Bio-Rad) (Gramaje et al., 2013; Morello et al., 2016).

#### Soil Isolation

We estimated the density of *V. dahliae* microsclerotia on the soil of both orchards at four times during the summer of 2012 and 2013. Microsclerotia were quantified using the wet sieving technique (Huisman and Ashworth, 1974) and the modified sodium polypectate agar medium (MSPA (Butterfield and Devay, 1977). In both orchards, the inoculum density was evaluated four times using 25-g-soil samples collected in June and October 2012 and 2013. Each 25-g-soil sample was constituted by 20–25 subsamples (approximately 1 g/sample) regularly collected in the studied orchards (Lopez-Escudero and Blanco-Lopez, 2007). The detection limit of the pathogen in the soil ranged from 0.2 to 0.02 microsclerotia per gram of soil, depending on the number of Petri dishes (0.25 g of soil/dish) used in each evaluation. Because of the low density of *V. dahliae* inoculum in the soil, we combined the whole of data to increase the limit of detection of the pathogen.

#### Pathotype Identification

Fifty *V. dahliae* isolates from 25 olive trees of each orchard were assigned to defoliating or non-defoliating pathotype by PCR using two couples of specific primers (DF/DR vs. NDF/NDR) (Perez-Artes et al., 2000).

### Disease Dynamic Through Digital Orthophotography

Digital color orthophotographs, dated in 1998, 2001, 2004, 2005, 2006, 2008, and 2010 at a 1:5,000 scale, were used to study the *Verticillium* wilt epidemics in both orchards. All digital orthophotographs were taken during July–August by the Instituto Cartografico de Andalucia, distributed by Red de Information Ambiental de Andalucia (REDIAM)^1^. The orthophotographs were processed using the gvSIG software (Valencian Generalitat Geographic Information System)^2^. The spatiotemporal dynamics of the *Verticillium* epidemic were based on the peer expert interpretation of the digital images. For that, we added a layer of points to each digital orthophotograph, in which each point represents the given position of the tree. Then, we generated a database for every given position with the following information: the binary mortality [alive (= 1) or death tree (= 0)] and absence/presence of tree. Because the projection of the crown size of a developing olive trees is associated with the years after plating and considering that the used images were ortho-rectified (REDIAM)^3^, the tree age was calculated according to its crown projection (Diaz-Varela et al., 2015). The Ancla orchard layer had 2,138-point olive trees, whereas the Granon orchard layer had 3,139-point olive trees (1664 cv. Picual and 1475 cv. Frantoio).

### Data Analysis

We differentiated the following treatments: olives of susceptible cv. Picual growing on Ancla orchard (treatment Picual–Ancla) and olives of moderately resistant cv. Frantoio and the susceptible cv. Picual growing on Granon orchard (treatments: Frantoio–Granon and Picual–Granon, respectively).

#### Field Data

Zar’s test of multiple comparisons of proportions was used for mean separation of the mortality (%) between treatments (Zar, 2010). Severity and DII data were analyzed using the non-parametric Kruskal–Wallis test, and mean ranks of the cultivars were compared at *P* = 0.05 using Dunn test with a Bonferroni adjustment at *P* = 0.05 (Demšar, 2006).

#### Orthophotograph Data

We stratified the data comparing the accumulated mortality at 8th and 10th years after planting for the three orchard-cultivar combinations to eliminate the planting year factor. Subsequently, we calculated the relative risk (RR, syn. risk ratio) associated with IDM by comparing the cv. Picual growing in both orchards and RR associated with the cultivar resistance by comparing both cultivars in Granon orchard. RRs were calculated dividing the mortalities of the treatments to compare, and χ^2^-tests were used to evaluate the significance of the RRs at *P* < 0.05 (Newman, 2001). These RRs are an optimum measure for both IDM and the use of the cultivar with different grade of resistance.

For each treatment, the increase in mortality (%) over time, considering the replanting of dead olive trees (i.e., several trees could die in a specific position throughout the study) and without considering it, was fitted using several linear and non-linear models. The best model was a Weibull model that includes rate and shape parameters, which allowed the generation of a wide variety of response curves according to the equation:

$$Y=a-b\,\left(\text{Exp}\,\left(-c\times t^{d}\right)\right)$$

where *Y* = accumulated mortality (%), *a* = upper asymptote parameter (i.e., upper limit of *Y*), *b* = an intrinsic rate of *Y* increase over time, *c* = a shape parameter (a higher *c* value, the function takes longer to reach the asymptote), *t* = time (year after planting), and *d* = a shape parameter associated with the type of curve. In all cases, the *R*^2^-values of the Weibull models were > 0.920, and standardized residuals were randomly distributed over predicted *Y* (Campbell and Madden, 1990). Subsequently, we compared the change rates (ΔY/Δt) between treatments using analysis of variance, followed by the least significant differences at *P* = 0.05, or *t*-test considering replanting or no-replanting. Scaled (standardized) area under disease progress curve was calculated by trapezoidal integration of mortality values over time divided by the duration of the observed epidemics (Madden et al., 2007). Survival analyses on the occurrence and timing after plantation of olive death were measured using mortality curves estimated by Kaplan–Meier method (Scherm and Ojiambo, 2004), and the treatments were compared using a log-rank test at *P* = 0.05 as previously for *Verticillium* epidemics (Perez-Rodriguez et al., 2016).

The relationship of the height above the sea level (m) and the accumulated olive tree mortality (%) were analyzed according to a logistic regression using the number of olive trees as a weight variable. Significance of the logistic regression was considered according to the *P*-value and regression coefficient (*R*^2^). The mortality (%) of olive trees of each year according to digital orthophotographs was correlated (Pearson correlation) with several weather variables from March of the previous year (n - 1) to June (n) to study the effect of weather variables on *Verticillium* wilt epidemics. For these correlations, we used the following weather variables: maximum, minimum, and average temperatures; and monthly and seasonal rainfall (mm) [fall (September–November), winter (December–February), spring (March–May), and total]. Data from this and the above experiment were analyzed with SPSS (version 14; SPSS Inc., Chicago, IL, United States) or Statistix 10 (Analytical Software, Tallahassee, FL, United States).

#### Spatial Analysis of Orthophotograph Data

Exponential kriging, an interpolation technique that was previously selected, was used to generate *Verticillium* wilt maps of both orchards according to the binary mortality data (Bailey, 1994). For that, a semivariance analysis followed by kriging was achieved by using the module “Sextante” of gvSIG software^4^. This interpolation technique has previously shown a good fit for *Verticillium* wilt data (Navas-Cortes et al., 2008). Spatial correlation between neighboring olive trees was conducted using the join-count statistic considering the largest square matrix in the center of each orchard (PASSgE, version 2.0; Rosenberg and Anderson, 2011).

Distance indices (SADIE) was used to study the strength and directionality or orientation of aggregation among quadrants containing dead olive trees by *Verticillium* wilt. For that, we calculated general clustering index (*Ia*) and clustering indices of patches (*V*_*i*_) and gaps (*V*_*j*_) using the SADIE software (Perry et al., 1996), in which the patches and gaps are considered areas with counts of dead trees greater and less than the mean, respectively (Perry et al., 1999). SADIE analysis was conducted on four and three subplots of the Granon and Ancla orchards, respectively (Supplementary Table S3).

Finally, the Index of Dispersion (*I*_β_) was calculated as the ratio between the observed variance (*V*_obs_) of the data between quadrants to the expected binomial variance (*V*_bin_). For that, we used an iterative process according to quadrant size (QSn; from *n* = 16 to 48) using a Visual Basic Excel 2007 macro (Microsoft Office, 2007), in which the hypothesis significance was calculated by χ^2^-test (*P* < 0.05) (Hughes and Madden, 1992; Madden and Hughes, 1995). Likewise, we calculated the *K*-value associated with the *I*_β_ as *K* = (*I*_β_ – 1)/(n – 1), where *n* is the quadrant size. *K*-value close to zero indicates a random spatial arrangement of the disease (Navas-Cortes et al., 2008), whereas positive values of K show its aggregation within a quadrant (Madden and Hughes, 1995). Finally, we fitted *Verticillium*-dead olive trees from both orchards to a beta (BD) and beta-binomial distributions (BBD), which indicate randomness or heterogeneity (aggregation or overdispersion), respectively, of the binary data of plant mortality (Madden and Hughes, 1995), using the software for MS-DOS, BBD.

## Results

### Field Evaluation

The impact of the IDM strategy on *Verticillium* wilt of olive was evaluated by comparing the disease on the susceptible olive cv. Picual, growing in two orchards, Ancla (traditionally managed with no IDM) and Granon (IDM). In Granon orchard, we also compared the *Verticillium* wilt epidemics of this cultivar and the moderately resistant Frantoio. Under field conditions (from June 2012 to October 2013), two Frantoio olive trees died due to the pathogen (0.60%), while the mortality of Picual olive trees in the Ancla orchard reached 41.5%. The mortality of Picual olives in Ancla orchard was significantly higher (*P* < 0.05) than the mortality (%) of Frantoio and Picual olives in Granon orchard, which did not show significant (*P* > 0.05) differences between them. Overall, through the study, significant differences (*P* < 0.05) were found in the incidence between treatments, as Picual–Ancla > Picual–Granon > Frantoio–Granon of trees died. Both Frantoio and Picual showed similar (*P* > 0.05) DII and severity in Granon orchard. Conversely, Picual olive trees showed lower (*P* < 0.05) DII and severity than them in Ancla orchard (Table 1).

**TABLE 1:** Mortality (%), incidence (%), Disease Intensity Index (DII), and severity of olive trees of the cvs. Picual (susceptible) and Frantoio (moderately resistant) affected with *Verticillium* wilt in two commercial orchards with different disease management practices in Southern Spain.

Orchard^*a*^/cv		June 2012				October 2012				April 2013			
Granon^*a*^	n	Mortality^*b*^	Incidence^*b*^	DII^*cd*^	Severity^*cd*^	Mortality	Incidence	DII	Severity	Mortality	Incidence	DII	Severity
Frantoio	336	0.30b	1.79a	0.04b	2.00b	0.30b	1.79a	0.04b	2.00b	0.30b	1.79a	0.04b	2.42b
Picual	257	1.17b	7.00b	0.24b	3.38ab	1.17b	7.00b	0.20b	2.91b	1.95b	8.95b	0.25b	2.75b

**Ancla**													
Picual	200	21.00a	38.50c	1.71a	4.44a	32.50a	42.00c	2.15a	5.12a	32.50a	45.00c	2.22a	4.93a

**Orchard/cv**		**June 2013**				**October 2013**							
**Granon**	**n**	**Mortality**	**Incidence**	**DII**	**Severity**	**Mortality**	**Incidence**	**DII**	**Severity**				

Frantoio	336	0.60b	1.79a	0.05b	2.75b	0.60b	2.08a	0.06b	2.71b				
Picual	257	3.11b	10.89b	0.31b	2.84b	3.11b	12.06b	0.33b	2.77b				

**Ancla**													
Picual	200	34.00a	45.50c	2.33a	5.12a	41.50a	60.00c	2.94a	4.90a				

**^*a*^Granon orchard was managed with an Integrated Disease Management (IDM) strategy by increasing planting density with cv. Frantoio, chemical weed control, and replantation of the dead trees with cv. Frantoio after soil solarization. Ancla orchard was traditionally managed by plowing and replanting dead trees with cv. Picual. ^*b*^Means with the same letter are not significantly different according to Zar’s test of comparison of proportions at P < 0.05. ^*c*^Means with the same letter are not significantly different according to Dunn all-pairwise comparisons after Kruskal–Wallis test at P < 0.05. ^*d*^Means of diseases severity (0–6) of all the evaluated (symptomatic and non-symptomatic) olives.**

The species *V. dahliae* was isolated in cultured media from 20% of the symptomatic olive samples belonging to cv. Picual, from both orchards, but the pathogen was detected by qPCR in the 76% of these samples. Regarding the moderately resistant cv. Frantoio, the pathogen was detected by qPCR only in the two dead olives. Molecular identification conducted using 25 *V. dahliae* isolates of each orchard showed the following rate among pathotypes (non-defoliating/defoliating): 2/23 (Ancla orchard) and 7/18 (Granon orchard) being this difference not significant (*P* = 0.066).

In 2002, the average densities of microsclerotia of *V. dahliae* per gram of soil were 0.4 and 0.5 in Ancla and Granon orchards, respectively (Moral, unpublished data). In 2012–2013, the averages of inoculum considering the four samplings were 0.040 and 0.018 microsclerotia per gram of soil in the Ancla and Granon orchards, respectively. According to these results, the density of *V. dahliae* propagules decreased around 10-fold (Ancla orchard) and 20-fold (Granon orchard) over 12–13 years.

### Disease Dynamic Through Digital Orthophotography

#### Temporal Analysis

**FIGURE 1:**
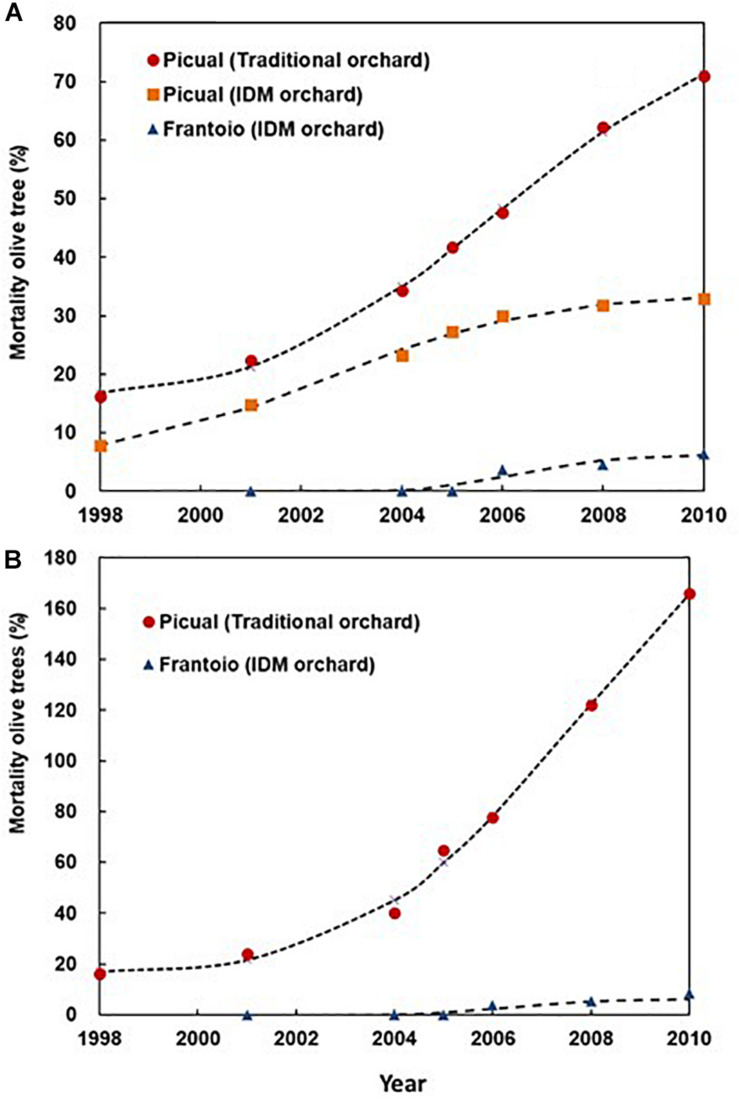
Accumulated mortality of olive trees cvs. Picual (susceptible) and Frantoio (moderately resistant) by *Verticillium dahliae* in two orchards in Southern Spain. In Granon orchard, an Integrated Disease Management of the *Verticillium* wilt was applied, while traditional agronomic (no-IDM) practices were applied in Ancla orchard. The lines represent the fitted curves according to a Weibull Model (Supplementary Table S2). **(A)** Accumulated olive tree mortality without considering plant replanting. **(B)** Accumulated olive tree mortality considering plant replanting.

**FIGURE 2:**
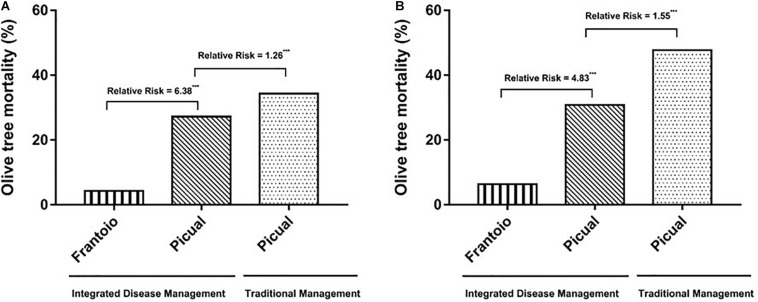
Accumulated mortality of olive trees cvs. Picual (susceptible) and Frantoio (moderately resistant) by *Verticillium dahliae* in two orchards in Southern Spain in the 8th **(A)** and 10th **(B)** years after plantation. In Granon orchard, an Integrated Disease Management (IDM) of the *Verticillium* wilt was applied, whereas traditional agronomic (no-IDM) practices were applied in Ancla orchard. Relative risk significant according to χ^2^-test at ***P < 0.001.

In 1998, we observed the first Picual olive trees with unequivocal symptoms of *Verticillium* wilt in both Ancla and Granon orchards. Two years later, the pathogen was isolated from 10 olive trees in each orchard. In the case of the moderately resistant cv. Frantoio, the first olive trees showing *Verticillium* wilt symptoms were observed in 2006, i.e., in year 6 after they were planted. Irrespective of replanting (i.e., considering that only one tree was planted in each position), the accumulated mortalities of Picual olive trees were 71.00 and 32.95% in Ancla and Granon orchards, respectively. In the case of the moderately resistant cv. Frantoio, the accumulated mortality was 6.37% (Figure 1). When we compared the mortality of the olive cv. Picual in both orchards, the RRs due to inadequate agronomic practices were 1.26 and 1.55 in years 8 and 10 after planting, respectively. Likewise, the susceptible cv. Picual, in contrast to the moderately resistant cv. Frantoio, was associated with an around fivefold increase in the mortality risk (χ^2^-test *P* < 0.001) (Figure 2). Considering the replantation, the accumulated number of dead Picual olive trees in the Ancla orchard was 3546 (165.86%), whereas it was 8.54% with Frantoio in the Granon orchard (Supplementary Table S1). Interestingly, Picual olives were planted even four times in 171 given positions in the Ancla orchard.

The Weibull model adequately (*R*^2^ > 0.900) described the increase in dead olives (with or without replanting) over time in both orchards. In all cases, there were significant differences (confidence interval at 95%) among the *a* and *b* parameters, i.e., upper asymptote and rate of the accumulated mortality, of the Weibull model for the three treatments; whereas no differences were observed among the shape parameters of the model (*c* and *d*) (Figure 1). Attending the average rate of change, there were significant differences (*P* = 0.001) among the epidemic rates being 5.59, 3.02, and 0.72% of *Verticillium*-dead olive per year for the treatments Picual–Ancla, Picual–Granon, and Frantoio–Granon without replanting, respectively. Likewise, the epidemic rate was 1.02% yr^–1^ for the treatment Frantoio–Granon, i.e., 13 times lower than Picual–Ancla (*P* = 0.008) (Supplementary Table S2). According to the Kaplan–Meier test, *Verticillium* wilt epidemics took 6 and 10 years to cause the death of 25% of olives cv. Picual in the Ancla and Granon orchards, respectively. Conversely, the pathogen caused a 6.37% of mortality of olives cv. Frantoio over 10 years. There were significant differences (Longrank test, *P* < 0.001) among the three treatments (Supplementary Figure S1).

During the study term, there was a significant (*P* < 0.05) and negative relationship between the number *Verticillium*-dead trees of the susceptible olive cv. Picual and the height above sea level. This relationship was significant (*P* < 0.05) in both orchards except for the combination Ancla 2004, it was clearer in the Granon orchard than in Ancla orchard (Table 2). Conversely, there was no significant relation between both mortality of the olive trees cv. Frantoio and height above sea level. Finally, we found positive regression (*R*^2^ = 0.961; *P* < 0.001) between total rainfall during March through May and the olive mortality in the Ancla orchard.

**TABLE 2:** Relationship between the height (m) above sea level and the accumulated mortality of olive trees cvs. Picual (susceptible) and Frantoio (moderately resistant) by *Verticillium dahliae* in two commercial orchards in Southern Spain.

			Logit model’s parameters			
Orchard^a^ Height (m)	Cultivar	Year	Constant	Height	*P*	*R*^2^
Ancla	Picual	1998	2.672	–0.117	0.001	0.220
260–312		2001	2.391	–0.010	0.001	0.203
		2004	0.953	–0.004	0.068	0.064
		2005	2.945	–0.011	< 0.001	0.238
		2006	2.969	–0.010	< 0.001	0.285
		2008	2.362	–0.007	0.001	0.188
		2010	2.217	–0.006	0.007	0.138
Granon	Picual	1998	16.840	–0.065	< 0.001	0.695
264–284		2001	13.966	–0.053	< 0.001	0.629
		2004	12.643	–0.047	< 0.001	0.574
		2005	13.476	–0.502	< 0.001	0.668
		2006	13.368	–0.049	< 0.001	0.623
		2008	14.906	–0.054	< 0.001	0.584
		2010	14.744	–0.054	< 0.001	0.581
Granon	Frantoio	2006	–8.789	0.026	0.098	0.211
264–284		2008	–3.635	–0.008	0.531	0.029
		2010	0.774	–0.007	0.528	0.022

**^*a*^Granon orchard was managed with an Integrated Disease Management (IDM) strategy by increasing planting density with the moderately resistant cv. Frantoio, chemical weed control, and replantation of the dead trees with cv. Frantoio after soil solarization. Ancla orchard was traditionally managed by plowing and replanting dead trees with cv. Picual. ^*b*^Logit model according to the equation Logit (P/1 - P) = Constant + b × height (m), where P = accumulated mortality. We used the number of olive trees at each height (m) as a weight variable**

#### Spatiotemporal Analysis

**FIGURE 3:**
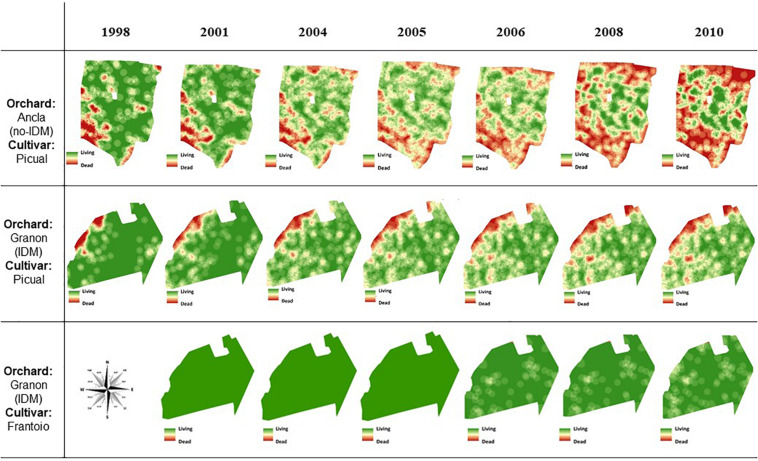
Kriging estimate maps based on *Verticillium*-dead mortality of olive trees in two commercial orchards in Southern Spain in the summer of each of 1998, 2001, 2004, 2005, 2006, 2008, and 2010 crop seasons. In Granon orchard, an Integrated Disease Management (IDM) of the *Verticillium* wilt has been applied since 1998. Olive trees of the susceptible cv. Picual were planted during fall–winter 1996–1997 in Granon orchard. In 2000, in the latter orchard, an olive tree of the resistant cv. Frantoio was planted in the middle of every four olive trees of the cv. Picual. Olive trees of the cv. Picual were planted in fall–winter 1995–1996 in Ancla orchard, in which traditional agronomic (no-IDM) practices have been applied since tree planting.

Figure 3 and Supplementary Figure S2 show the spatiotemporal pattern of the mortality caused by *V. dahliae* on both orchards. In 1998, olives of the cv. Picual at the southwestern side of the Ancla orchard and the northwestern side of the Granon orchard, respectively, were the most affected by the pathogen. In the Ancla orchard, the size of the principal focus increased over time, whereas new foci mainly developed at the edges, and only a central area of the orchard was *Verticillium*-free. In the Granon orchard, the *Verticillium* epidemic affected the entire band of olive cv. Picual at the north side of the orchard at the end of this study. Considering the cv. Frantoio, small foci were observed without an apparent pattern in the sixth years after planting, but new wilt trees appeared more frequently next to original foci of *Verticillium*-dead olives (Figure 3). At the Granon orchard, the replacement of the dead trees of the susceptible cv. Picual with the moderately resistant cv. Frantoio decreased the aggregation of the disease. Because a higher aggregation of *Verticillium*-dead olive in the edges of both orchards and the edges were not considered for the join-count statistic because of limitation of the used software, this only showed significant aggregation between dead olive trees (*P* < 0.05) cv. Frantoio in Granon orchard (from 2006 to 2010) and cv. Picual (*P* < 0.001) in Ancla orchard in 2010 (data not shown).

Distance indices analysis (SADIE) was conducted in both orchards using different plots with 200–480 olives each. The clustering index (*I*_*a*_) and clustering indices of patches (*V*_*i*_) and gaps (*V*_*j*_) were correlated. The three SADIE indices were variable among plots and years. In Ancla orchard, we observed a significant aggregated pattern (*P* < 0.05) in at least one plot during the 12 years of this study. There was a reduction in the degree of aggregation of the disease through the time in the susceptible cv. Picual in the Granon orchard; thus, while the dead-*Verticillium* olives cv. Picual were aggregated, at least in one plot at the beginning of this study (1998 and 2001), the disease showed a random pattern at the end of this study (2008 and 2010). Conversely, once the first moderately resistant olives cv. Frantoio died in 2006, there was a significant aggregation of the *Verticillium* wilt according to *I*_*a*_, *V*_*i*_, and *V*_*j*_ (Supplementary Table S3). Overall, the replanting of dead olives decreased the heterogeneity of the *Verticillium*-dead olives in both orchards (data not shown).

Similar results were independently obtained of quadrant size. Thereafter, *I*_β_ was calculated considering a quadrant size of 48 trees. Thus, when *Verticillium*-dead olives showed a non-random pattern according to the *I*_β_ (*P* < 0.05), data significantly fitted to the BBD but not to BD. Results of the D and BBD analysis suggested an aggregated pattern of distribution of *Verticillium*-dead olive trees of the susceptible cv. Picual in both orchards through the 12 years. Likewise, in Granon orchard, *Verticillium*-dead olives of the moderately resistant cv. Frantoio showed a heterogeneous pattern, but only in 2006 and 2008 (Table 3). In Ancla orchard, there was an important decline of the *I*_β_ from 1998 to 2005 because of the increase in the dead trees, but it subsequently remained stable around 5.5. Likewise, in Granon orchard, the *I*_β_ suggested aggregation of *Verticillium*-dead olives cv. Picual and a light aggregation of the dead olives cv. Frantoio, given the low number of dead plants. Besides, as expected, the replanting process decreased the aggregation values (D and BBD) of the disease (data not shown).

**TABLE 3:** Index of Dispersion (*I*_β_) and probability to no-fitting binomial (BD) and fittings beta-binomial (BBD) of the distributions of dead olive trees cvs. Picual (susceptible) and Frantoio (moderately resistant) by *Verticillium dahliae* in two commercial orchards with different disease management practices in Southern Spain.

Orchard cultivar	1998				2001				2004				2005			
				
Granon^a^	*I*_β_^b^	*P*^b^	BD-χ (*P*)^c^	BBD-*z* (*P*)^d^	*I*_β_	*P*	BD-χ (*P*)	BBD-*z* (P)	*I*_β_	*P*	BD-χ (*P*)	BBD-*z* (*P*)	*I*_β_	*P*	BD-χ (*P*)	BBD-*z* (*P*)
Picual	4.55	< 0.001	<0.001	< 0.001	4.40	< 0.001	<0.001	< 0.001	2.83	< 0.001	0.001	< 0.001	2.61	< 0.001	0.230	< 0.001

**Ancla**^a^																
Picual	9.25	< 0.001	0.025	< 0.001	10.70	< 0.001	0.001	< 0.001	6.10	< 0.001	0.001	< 0.001	5.34	< 0.001	0.018	< 0.001

**Orchard cultivar**	**2006**				**2008**				**2010**							
				
**Granon**	***I*_β_**	***P***	**BD-χ (*P*)**	**BBD-*z* (*P*)**	***I*_β_**	***P***	**BD-χ (*P*)**	**BBD-*z* (*P*)**	***I*_β_**	***P***	**BD-χ (*P*)**	**BBD-*z* (*P*)**				

Picual	2.71	< 0.001	0.034	< 0.001	2.83	< 0.001	0.227	< 0.001	2.87	< 0.001	0.486	< 0.001				
Frantoio	1.33	0.054	0.025	0.053	1.38	0.035	0.048	< 0.001	1.30	0.073	0.340	0.075				

**Ancla**																
Picual	5.87	< 0.001	0.002	< 0.001	5.10	< 0.001	<0.001	< 0.001	5.49	< 0.001	0.002	< 0.001				

**^*a*^Granon orchard was managed with an Integrated Disease Management (IDM) strategy by increasing planting density with cv. Frantoio, chemical weed control, and replantation of the dead trees with cv. Frantoio after soil solarization. Ancla orchard was traditionally managed by plowing and replanting dead trees with cv. Picual. ^*b*^A large Index of Dispersion (I_β_) > 1 combined with a small probability (P < 0.05) suggests aggregation of Verticillium-dead olive trees. ^*c*^Probability no-fitting to binomial (BD) distribution (i.e., randomness of Verticillium-dead olive trees) according to χ^2^*-*test. ^*d*^Probability fitting to beta-binomial (BBD) distribution (i.e., aggregation of Verticillium-dead olive trees) according to standard z-value.**

## Discussion

*Verticillium* wilt is the most important disease of olive in many countries of the Mediterranean basin, having a major impact in Spain, the main olive producer worldwide (Lopez-Escudero and Mercado-Blanco, 2011; Jimenez-Diaz et al., 2012). Control of *Verticillium* wilt of olive depends heavily on the use of resistant cultivars; however, there are no completely resistant cultivars (immune) to the pathogen (Lopez-Escudero et al., 2004; Leyva-Perez et al., 2017). The use of moderately resistant olive cultivars to *V. dahliae* should be applied in a coordinated manner with other management disease practices to maximize the benefits of this resistance. Here, we studied the effect that an IDM performed in an orchard (called Granon) of the susceptible olive cv. Picual in the spatiotemporal development of *Verticillium* wilt by comparing with a traditional orchard (called Ancla). This IDM strategy comprised the increasing planting density with moderately resistant cv. Frantoio, chemical weed control, and replanting of the dead tree with cv. Frantoio after soil solarization. Other studies suggest the replacement of wilted olive trees with apple trees, a non-host plant, as an environmentally friendly management practice (Karajeh and Owais, 2012), but little is known about replacement using resistant cultivars of olive.

Generally, the rate of *Verticillium*-dead olive trees per year was 5.59%, when the susceptible cv. Picual was growing in the orchard with no disease management practices, and 3.02% yr^–1^ when the same cultivar was at the IDM orchard. When planted in soils with low inoculum densities (<1 microsclerotia/g of soil), the cv. Picual is extremely susceptible to the defoliating pathotype of *V. dahliae* (Lopez-Escudero and Blanco-Lopez, 2007). When the IDM was performed in orchards planted with the moderately resistance cv. Frantoio, the annual rate of dead olive trees was around sevenfold lower than the observed in the orchard more conducive for the disease, i.e., susceptible cultivar and non–disease management practices. Likewise, according to the RR, the impact of using a moderately resistant cultivar in the reduction of tree mortality was much higher than the application of different agronomic practices between orchards. It is worth mentioning here that two agronomic factors could have favored the epidemic development in Granon orchard. On one side, irrigation water disseminates the pathogen through the olive orchards of Southern Spain (Garcia-Cabello et al., 2012) (Ancla was rainfed during the duration of the study) and increases the olives susceptibility to pathogen infection (Perez-Rodriguez et al., 2016; Santos-Rufo et al., 2017). Second, the plant density, when higher because of the interplanting of the moderately resistant cv. Frantoio, reduced the distance between adjacent trees and increased the potential number of infected olives and, simultaneously, the inoculum source. Roca et al. (2016) observed that non-significant effect of the plating density on the epidemic of *Verticillium* wilt of olive was not observed, although this study was conducted on the cvs. Arbequina and Picual.

The differences in susceptibility to *V. dahliae* between cvs. Frantoio (syn. Oblonga) and Picual have been well-known for a long time (Hartmann et al., 1971; Lopez-Escudero et al., 2004). The moderate resistance of the cv. Frantoio is associated with a protein of Bet v I family and a dirigent-like protein involved in lignification and reactive oxygen species stress response (Leyva-Perez et al., 2017). The phenotypic reaction of the cv. Frantoio to the pathogen is well-known under controlled conditions and experimental fields (Lopez-Escudero et al., 2004; Gramaje et al., 2013; Trapero et al., 2013). Unfortunately, few or too short-term studies have been conducted in naturally infested soils (Montes-Osuna and Mercado-Blanco, 2020). For example, Trapero et al. (2013) observed no affected olives of the cv. Frantoio in highly infested soils (21 microsclerotia/g) but only in a 2-year study. Under the conditions of our study, the first *Verticillium* dead tree of the cv. Frantoio was detected in the sixth year after planting, once the mortality had reached up to 4.27% of the trees of this cultivar, whereas susceptible Picual showed mortality around 30%.

In our study, and although there was no periodic monitoring, there was a 20-fold decrease from 2000 to 2012–2013 in the density of *V. dahliae* microsclerotia in Granon orchard. This reduction may be because of the immediate burning of fallen leaves of the olive trees affected by the pathogen, which are the major source of new microsclerotia (Jimenez-Diaz et al., 2012). However, in the Ancla orchard, an important decrease (around 10-fold) in the microsclerotia density was also detected. This observation suggests that the balance between microsclerotia production and their loss decreases the inoculum in soil, but more studies are needed. Conversely, in several herbaceous hosts, when affected by *V. dahliae*, a marked increment of inoculum in the soil is observed at the end of the season (Mol and Termorshuizen, 1995; Ioannou et al., 1977).

In the rainfed orchard, the disease epidemic was driven by the accumulated rainfall during the spring, which is in accordance with the fact that the maximum infection rate of *Verticillium* wilt of olive occurs at the end of the winter–early spring (Navas-Cortes et al., 2008) and favored by moist soils (Perez-Rodriguez et al., 2016; Santos-Rufo et al., 2017).

The disease cycle, among other factors, such as the cultivar resistance, the agronomic practices, and the climatic variable of the region, determines the spatial pattern of the diseases (Madden et al., 2007). Factors such as the spatial distribution of the microsclerotia (Bejarano-Alcazar et al., 1995; Xiao et al., 1997) and the potential use of infected plating material (Thanassoulopoulos, 1992) have an essential impact on the spatial pattern of the *Verticillium* wilt of olive during the first years after planting. After this, *V. dahliae* spreads between, and within, olive orchards through infested soil particles and machinery, irrigation water, and infected plant debris, and thus affecting the spatial pattern of the disease (Lopez-Escudero and Mercado-Blanco, 2011; Jimenez-Diaz et al., 2012). In our study, we observed an aggregated pattern of *Verticillium*-dead olives in both orchards, Ancla and Granon, although a lower level of aggregation in the latter. The height (m) of the trees explained part of this aggregation since the disease leaned to cluster in the low-lying parts of the orchards as a consequence of higher inoculum density. Similarly, the *Verticillium* wilt epidemics of lettuce usually start in low-lying fields/areas in California (Vallad et al., 2006). In any case, results shown in the current study agree with many diseases caused by soil-borne pathogens, including the *Verticillium* wilt of olive (Navas-Cortes et al., 2008), tending to be more aggregated than aerial pathogens (Campbell and Benson, 1994).

Finally, we also observed that the replanting of dead olives decreased aggregation of the disease, mainly when conducted employing the moderately resistant cv. Frantoio. Furthermore, the level of disease aggregation decreased with the accumulated mortality of olive trees because of the homogenization process of the disease in the field (Turechek et al., 2011).

Under the conditions of our study, which include low inoculum pressure of *V. dahliae* and low winter temperatures that reduce the period of infection (Trapero et al., 2013), the described IDM strategy has allowed an acceptable control of the *Verticillium* wilt and has kept the profitability of the Granon orchard.

## Data Availability Statement

The raw data supporting the conclusions of this article will be made available by the authors, without undue reservation, to any qualified researcher.

## Author Contributions

JM conceived and designed the study. JM, EO, and FL-E performed the evaluation under field conditions. EO and MG-L performed the laboratory works. JM, RP, and EO analyzed the data. JM, MG-L, and TM wrote the manuscript. FL-E, AT, RP, and EO reviewed and discussed the final revised version. All authors contributed to the article and approved the submitted version.

## Conflict of Interest

The authors declare that the research was conducted in the absence of any commercial or financial relationships that could be construed as a potential conflict of interest. The reviewer FJGG declared a shared affiliation, with no collaboration, with several of the authors, EO, MG-L, FL-E, AT-C, JM, to the handling editor at the time of the review.

## Footnotes

1 http://www.juntadeandalucia.es/medioambiente/site/rediam

2 http://www.gvsig.com/

3 http://www.juntadeandalucia.es/medioambiente/site/rediam

4 http://www.gvsig.com/

## Supplementary Material

Supplementary Figure 1 Kaplan-Meier survival functions of olive trees cvs. Picual (susceptible) and Frantoio (moderately resistant) affected by *Verticillium dahliae* in two orchards in Southern Spain. In Granon orchard, an Integrated Diseases Management (IDM) of the *Verticillium* wilt was applied, while traditional agronomic (no-IDM) practices were maintained in Ancla orchard.

Supplementary Figure 2 Orthophotographs showing *Verticillium*-dead mortality of olive trees in two commercial orchards in Southern Spain in the summer of each of 2001, 2006, and 2010 crop seasons. In Granon orchard, an Integrated Diseases Management (IDM) of the *Verticillium* wilt was applied since 1998. Olive trees of the susceptible cv. Picual were planted during fall-winter 1996–1997 in Granon orchard. In 2000, in the latter orchard, an olive tree of the resistant cv. Frantoio was planted in the middle of every four olive trees of the cv. Picual. Olive trees of the cv. Picual were planted in fall-winter 1995–1996 in Ancla orchard, in which traditional agronomic (no-IDM) practices were applied since tree planting.
