# Supplementary material for: Effect of Cultivar Resistance and Soil Management on Spatial–Temporal Development of Verticillium Wilt of Olive: A Long-Term Study
Source: Front Plant Sci. 2020 Oct 27;11:584496. doi: 10.3389/fpls.2020.584496 (PMC7652988; doi:10.3389/fpls.2020.584496)
Supplement: Supplementary file 1 [file Data_Sheet_1.docx]

| **Table S1.** Mortality (%) of olive trees of the cvs. Picual (susceptible) and Frantoio (moderately resistant) by *Verticillium dahliae* in two commercial orchards with different disease management practices in Southern Spain. | | | | | | | | | | |
| --- | --- | --- | --- | --- | --- | --- | --- | --- | --- | --- |
| **Year** | | | | | | | | | | |
| **Orchard^a^ /**  **No replanting** | **Cultivar** | **No** | **1998^a^** | **2001^a^** | **2004^a^** | **2005^a^** | **2006^a^** | **2008^a^** | **2010^a^** | **No Dead trees (%)** |
| **Granon** | **Picual** | 1663 | 7.70 | 7.04 | 8.42 | 4.09 | 2.65 | 1.92 | 1.14 | 548 (32.95) |
| **Granon** | **Frantoio** | 1475 | - | 0.00 | 0.00 | 0.00 | 3.73 | 0.81 | 1.83 | 94 (6.37) |
| **Ancla** | **Picual** | 2138 | 16.18 | 6.17 | 11.97 | 7.44 | 5.89 | 14.55 | 8.79 | 1518 (71.00) |
| **Mortality (No Tree)** |  |  | 474 | 249 | 396 | 227 | 225 | 355 | 234 | 2194 |
| **Orchrad /**  **With Replanting** | **Cultivar** | **No** | **1998^a^** | **2001^a^** | **2004^a^** | **2005^a^** | **2006^a^** | **2008^a^** | **2010^a^** | **No Dead trees (%)** |
| **Granon** | **Frantoio** | 1475 | - | 0.00 | 0.00 | 0.00 | 3.73 | 1.63 | 3.19 | 126 (8.54) |
| **Ancla** | **Picual** | 2138 | 16.18 | 7.76 | 16.32 | 24.51 | 13.10 | 44.06 | 43.92 | 3546 (165.86) |
| **Mortality (No Tree)** |  |  | 474 | 286 | 543 | 663 | 421 | 1092 | 1075 | 4554 |
| ^a^Granon orchard was managed with an Integrated Disease Management (IDM) strategy by increasing planting density with cv. Frantoio, chemical weed control, and replantation of the dead trees with cv. Frantoio after soil solarization. Ancla orchard was traditionally managed by plowing and replanting dead trees with cv. Picual | | | | | | | | | | |

| **Table S2.** Parameters and their statistical significance of the Weibull models for Verticillium wilt epidemics in two olive orchards with different disease management practices in Southern Spain | | | | | | | | | | | |
| --- | --- | --- | --- | --- | --- | --- | --- | --- | --- | --- | --- |
|  |  |  | ***Adjusted model*** | | | | |  |  |  |  |
| **Orchard^a^** | **Cultivar** | **Replanting** | **a^b^** | **b** | **c** | **d** | ***R^2^*** | **Inflection**  **Point (yr)** | **Max. Rate**  **(%yr^-1^)^c^** | **Avg. Rate** **(%yr^-1^)^d^** | **Scaled**  **AUDPC^e^** |
| **Granon** | **Picual** | **No** | 33.624b | 26.166b | 0.015a | 2.177b | 0.996 | 6 | 3.41 | 3.02b | 21.12 |
| **Granon** | **Frantoio** | **No** | 6.125c | 6.534c | 3×10^-4^a | 4.282b | 0.908 | 7 | 1.53 | 0.72c | 2.11 |
| **Ancla** | **Picual** | **No** | 79.809a | 63.312a | 5×10^-4^a | 3.144b | 0.999 | 10 | 6.85 | 5.59a | 34.63 |
| **Granon** | **Frantoio** | **Yes** | 9.304a | 9.708a | 4×10^-5^a | 3.825a | 0.946 | 8 | 1.77 | 1.02b | 2.48 |
| **Ancla** | **Picual** | **Yes** | 213.633b | 196.653b | 4×10^-5^a | 3.942a | 0.994 | 13 | 22.43 | 13.96a | 56.01 |
| ^a^Granon orchard was managed with an Integrated Disease Management (IDM) strategy by increasing planting density with the moderately resistant cv. Frantoio, chemical weed control, and replantation of the dead trees with cv. Frantoio after soil solarization. Ancla orchard was traditionally managed by plowing and replanting dead trees with cv. Picual  ^b^Weibull model according to the equation Y =a-b×Exp(-c×t^d^) where Y = accumulated mortality and t = years after planting  ^c^Maximum rate (% yr^-1^) of Verticillium-dead olive calculated according to the adjusted Weibull model  ^d^Average rate (% yr^-1^) of Verticillium-dead olive calculated according to the mortality data. Means with the same letter are not significant different according to Least Significant Differences at *P =* 0.05.  ^e^Scaled (standardized) Area Under Disease Progress Curve was calculated according to Madden et al. (2007). | | | | | | | | | | | |

| **Table S3.** Spatial analysis by distance indices (SADIE) of the tree mortality by Verticillium wilt in two olive orchards with different disease management practices in Southern Spain | | | | | | | | | | | | | | |
| --- | --- | --- | --- | --- | --- | --- | --- | --- | --- | --- | --- | --- | --- | --- |
| **Orchard**  **Cultivar** |  |  | **1998** | | | **2001** | | | **2004** | | |  |  |  |
|  |  |  |  |  |  |  |  |  |  |  |  |  |  |  |
| **Granon^a^** | **Subplot** | **N** | **Ia^a^** | **Vj** | **Vi** | **Ia** | **Vj** | **Vi** | **Ia** | **Vj** | **Vi** |  |  |  |
| **Picual** | **1** | 240 | 2.020*** | -1.982*** | 2.031*** | 1.954*** | -2.008*** | 1.957*** | 0.95 | -0.964 | 0.947 |  |  |  |
|  | **2** | 240 | 1.042 | -1.006 | 1.053 | 0.965 | -0.957 | 0.974 | 0.981 | -0.971 | 0.988 |  |  |  |
|  | **3** | 264 | 1.984** | -2.013** | 1.998*** | 0.899 | -0.995 | 0.881 | 0.967 | -0.948 | 0.988 |  |  |  |
|  | **4** | 255 | 1.150 | -1.175 | 1.153 | 1.150 | -1.175 | 1.153 | 1.015 | -1.010 | 1.014 |  |  |  |
| **Frantoio** | **1** | 240 |  |  |  |  |  |  |  |  |  |  |  |  |
|  | **2** | 240 | ***NO DEAD OLIVE TREES*** | | | | | | | | |  |  |  |
|  | **3** | 216 |  |  |  |  |  |  |  |  |  |  |  |  |
|  | **4** | 216 |  |  |  |  |  |  |  |  |  |  |  |  |
| **Ancla** |  |  |  |  |  |  |  |  |  |  |  |  |  |  |
| **Picual** | **1** | 476 | 1.495** | -1.451** | 1.502** | 1.270 | -1.248 | 1.273* | 1.152 | -1.143 | 1.150 |  |  |  |
|  | **2** | 266 | 1.003 | -1.014 | 1.007 | 2.704*** | -2.718*** | 2.559*** | 2.519*** | -2.529*** | 2.343*** |  |  |  |
|  | **3** | 200 | 1.499 | -1.435 | 1.519* | 1.701* | -1.627* | 1.699* | 0.863 | -0.854 | 0.856 |  |  |  |
|  |  |  | **2005** | | | **2006** | | | **2008** | | | **2010** | | |
|  |  |  |  |  |  |  |  |  |  |  |  |  |  |  |
| **Granon^a^** | **Subplot** | **N** | **Ia^b,c^** | **Vj^c^** | **Vi^c^** | **Ia** | **Vj** | **Vi** | **Ia** | **Vj** | **Vi** | **Ia** | **Vj** | **Vi** |
| **Picual** | **1** | 240 | 0.952 | -0.958 | 0.947 | 1.051 | -1.054 | 1.044 | 1.050 | -1.052 | 1.044 | 1.113 | -1.120 | 1.106 |
|  | **2** | 240 | 0.879 | -0.874 | 0.882 | 0.935 | -0.929 | 0.94 | 0.908 | -0.901 | 0.913 | 0.938 | -0.929 | 0.944 |
|  | **3** | 264 | 1.976** | -2.009** | 1.971** | 1.563* | -1.556* | 1.571* | 1.188 | -1.185 | 1.193 | 1.162 | -1.114 | 1.172 |
|  | **4** | 255 | 0.992 | -0.996 | 0.991 | 0.969 | -0.973 | 0.969 | 0.97 | -0.97 | 0.97 | 0.98 | -0.981 | 0.98 |
| **Frantoio** | **1** | 240 |  |  |  | 0.982 | -0.974 | 0.986 | 0.982 | -0.974 | 0.986 | 1.023 | -1.018 | 1.028 |
|  | **2** | 240 | ***NO DEAD OLIVE TREES*** | | | 1.566* | -1.577* | 1.580* | 1.755** | -1.794** | 1.771** | 1.752** | -1.810** | 1.756** |
|  | **3** | 216 |  |  |  | 1.162 | -1.169 | 1.174 | 1.173 | -1.167 | 1.192 | 1.282 | -1245 | 1.299 |
|  | **4** | 216 |  |  |  | 1.389* | -1.445* | 1.383* | 1.389* | -1.445* | 1.383* | 1.301 | -1.338* | 1.303 |
| **Ancla** |  |  |  |  |  |  |  |  |  |  |  |  |  |  |
| **Picual** | **1** | 476 | 1.335* | -1.334* | 1.332* | 1.589** | -1.587*** | 1.587*** | 1.921*** | -1.928*** | 1.918*** | 1.847*** | -1.849*** | 1.844*** |
|  | **2** | 266 | 2.437*** | -2.448*** | 2.256*** | 2.437*** | -2.448*** | 2.256*** | 2.304*** | -2.312*** | 2.127*** | 2.262*** | -2.275*** | 2.103*** |
|  | **3** | 200 | 1.039 | -1.037 | 1.030 | 0.739 | -0.756 | 0.742 | 1.282 | -1.335 | 1.251 | 1.495* | -1.565* | 1.435* |
| ^a^Granon orchard was managed with an Integrated Disease Management (IDM) strategy by increasing planting density with the moderately resistant cv. Frantoio, chemical weed control, and replantation of the dead trees with cv. Frantoio after soil solarization. Ancla orchard was traditionally managed by plowing and replanting dead trees with cv. Picual  ^b^Analysis was performed using SADIE software (Rothamsted Research Institute, Rothamsted, UK).  ^c^*Ia* is the overall index of aggregation; Vi and Vj correspond to the average values of the indices of clustering Vi (patch of living olives) and Vj (gap of dead olives) | | | | | | | | | | | | | | |
