# Supplementary figures and images for: Effect of Cultivar Resistance and Soil Management on Spatial–Temporal Development of Verticillium Wilt of Olive: A Long-Term Study
Source: Front Plant Sci. 2020 Oct 27;11:584496. doi: 10.3389/fpls.2020.584496 (PMC7652988; doi:10.3389/fpls.2020.584496)

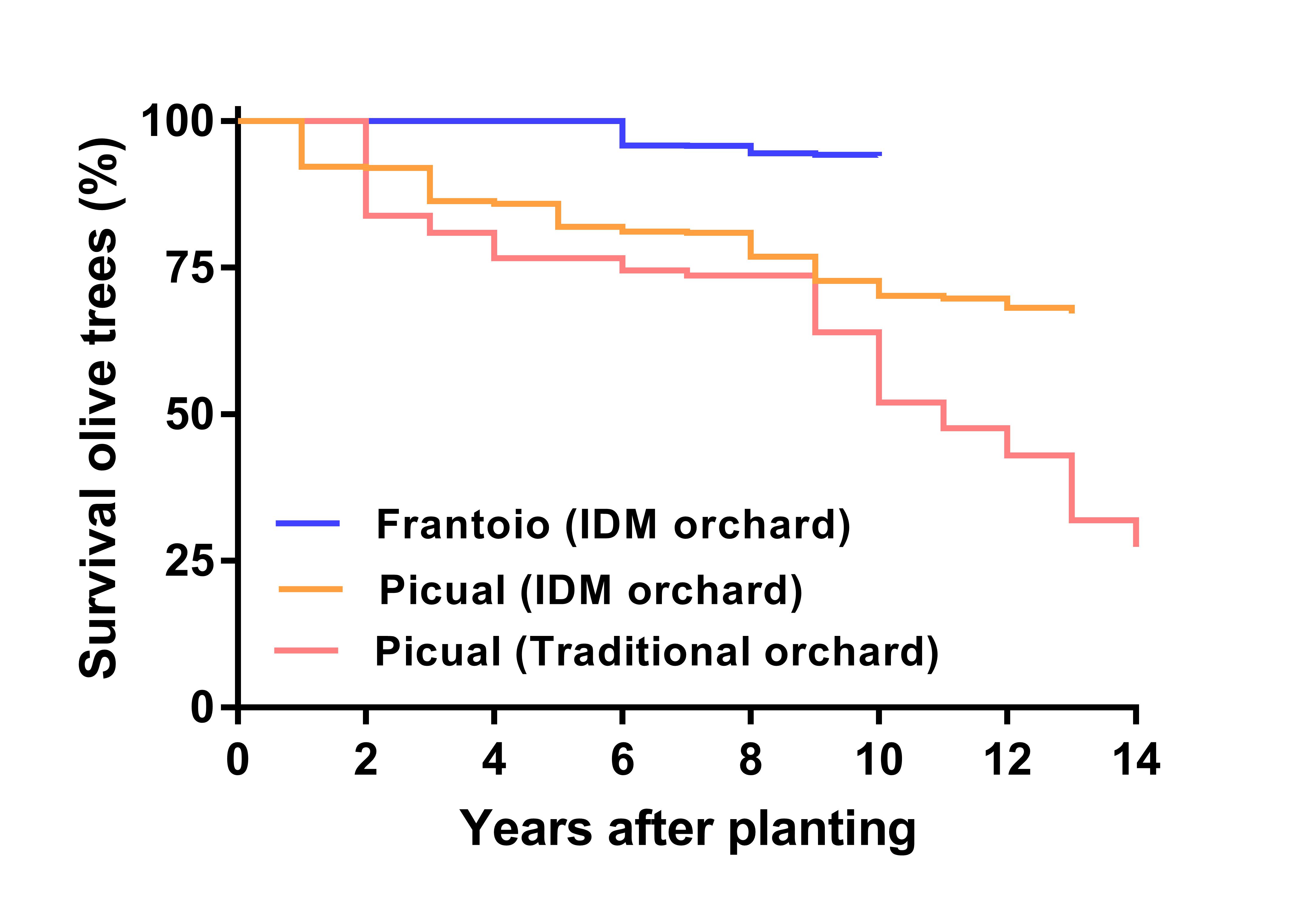

Supplement: Supplementary Figure 1 — Kaplan-Meier survival functions of olive trees cvs. Picual (susceptible) and Frantoio (moderately resistant) affected by Verticillium dahliae in two orchards in Southern Spain. In Granon orchard, an Integrated Diseases Management (IDM) of the Verticillium wilt was applied, while traditional agronomic (no-IDM) practices were maintained in Ancla orchard. [file Image_1.JPEG]

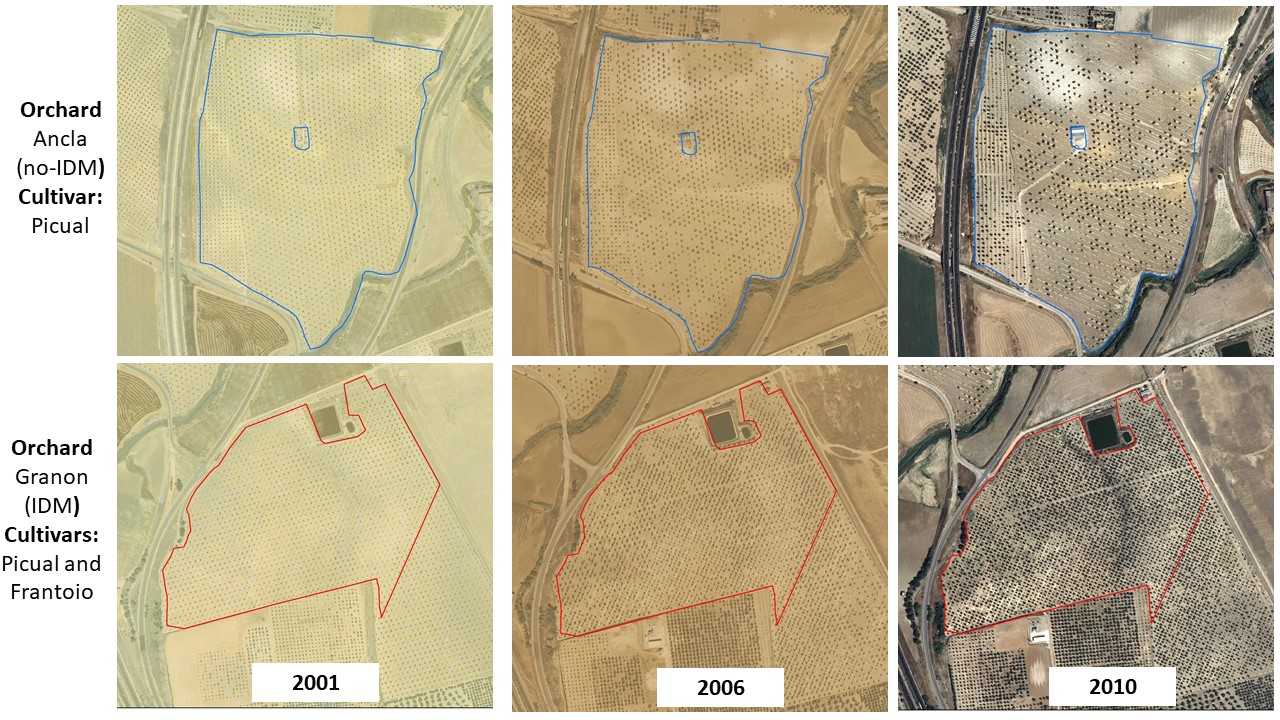

Supplement: Supplementary Figure 2 — Orthophotographs showing Verticillium-dead mortality of olive trees in two commercial orchards in Southern Spain in the summer of each of 2001, 2006, and 2010 crop seasons. In Granon orchard, an Integrated Diseases Management (IDM) of the Verticillium wilt was applied since 1998. Olive trees of the susceptible cv. Picual were planted during fall-winter 1996–1997 in Granon orchard. In 2000, in the latter orchard, an olive tree of the resistant cv. Frantoio was planted in the middle of every four olive trees of the cv. Picual. Olive trees of the cv. Picual were planted in fall-winter 1995–1996 in Ancla orchard, in which traditional agronomic (no-IDM) practices were applied since tree planting. [file Image_2.JPEG]
